# Treg/Th17 Cell Balance in Patients with Hepatitis B Virus-Related Acute-on-Chronic Liver Failure at Different Disease Stages

**DOI:** 10.1155/2021/9140602

**Published:** 2021-11-26

**Authors:** Nian-Hua Tan, Bin Chen, Jie Peng, Shan Du

**Affiliations:** Department of Hepatology, The First Affiliated Hospital of Hunan University of Chinese Medicine, Changsha, 410007 Hunan Province, China

## Abstract

**Background:**

T-helper 17 (Th17) and CD4^+^CD25^+^ T-regulatory (Treg) cells play important roles in the pathogenesis of hepatitis B virus-related acute-on-chronic liver failure (HBV-ACLF). This study is aimed at investigating shifts in Treg/Th17 balance in the peripheral blood of HBV-ACLF patients at different disease stages.

**Methods:**

Sixty HBV-ACLF patients, admitted to the First Hospital of Hunan University of Chinese Medicine, China, including early-stage (*n* = 20), middle-stage (*n* = 20), and late-stage patients (*n* = 20), were enrolled in the study. In addition, 20 patients with chronic hepatitis B and 20 healthy volunteers were also included in the study as controls. Flow cytometry, cytometric bead array, and quantitative real-time PCR protocols were used to evaluate the expression of Treg and Th17 cells as well as of related cytokines.

**Results:**

The levels of Th17 cells and their effectors interleukin- (IL-) 17A, IL-23, and tumor necrosis factor-*α* increased with disease progression. Similarly, Treg cells and their effector cytokines transforming growth factor-*β* and IL-10 also increased. Although Treg and Th17 levels were positively correlated, the latter were always at higher numbers. Noteworthy, the Treg/Th17 ratio gradually decreased and was negatively correlated with ACLF severity. *FoxP3* levels in the peripheral blood gradually decreased with ACLF progression, whereas *ROR-γt* gradually increased. Serum c-reactive protein, procalcitonin, and lipopolysaccharide were also upregulated with disease progression and positively correlated with Th17 abundance. Further, Th17, IL-17A, and IL-23 were independent risk factors for ACLF. A prognostic model for HBV-ACLF was established, with a correct prediction rate of 90.00% (54/60).

**Conclusion:**

Treg/Th17 imbalance occurs throughout the pathogenic course of HBV-ACLF, with an imbalance shift toward Th17. Hence, the Th17-mediated inflammatory response drives HBV-ACLF-associated inflammation and supports the pathological mechanisms of liver failure.

## 1. Introduction

Acute-on-chronic liver failure (ACLF) is a clinical condition characterized by an exacerbation of acute jaundice and coagulation dysfunction caused by various factors on the basis of chronic liver disease [1, 2]. ACLF progresses rapidly and has a high short-term mortality rate. Based on the severity of clinical symptoms, the disease course can be divided into early, middle, and late stages. In China, hepatitis B virus- (HBV-) related ACLF is the most common type of liver failure, accounting for over 80% of all liver failure cases [3]. The pathogenesis of HBV-ACLF is a complex immunopathological process, which remains poorly known. A growing body of evidence suggests that persistent inflammatory response and immune dysregulation are the core mechanisms underlying HBV-ACLF [4, 5]. An antiviral inflammatory response is observed in the early and advanced stages of the disease, followed by endotoxin-driven inflammation during the recovery stage, which drives an imbalance of anti- and proinflammatory factors that are closely related to prognosis [6].

CD4^+^ T cells play a central role in the adaptive immune response during liver failure. In particular, T-helper 17 (Th17) and T-regulatory (Treg) cells modulate the inflammatory response in liver failure; thus, these cell populations have become core research targets [7]. Tregs express the transcription factor forkhead box protein 3 (FoxP3) and mainly secrete interleukin- (IL-) 10, transforming growth factor-*β* (TGF-*β*), and IL-35, which are anti-inflammatory cytokines. Th17 cells express the transcription factor retinoid orphan receptor *γ*t (ROR-*γ*t) and mainly secrete IL-17A, IL-23, tumor necrosis factor-*α* (TNF-*α*), and IL-6, which are proinflammatory [8]. Thus, Treg and Th17 cells have opposing functions. However, they are also interdependent and regulate each other. Several clinical studies have shown that the Treg/Th17 balance is associated with the severity of HBV-ACLF and that Treg/Th17 dysregulation can reflect aberrant immune function in these patients. Moreover, increased or decreased levels of associated cytokines are related to the degree of liver injury, thus having a guiding role in clinical prognosis [9–15]. However, there is no consensus on the specific Treg/Th17 ratio changes observed in the peripheral blood of HBV-ACLF patients.

Therefore, in the present study, we determined the frequency of Treg/Th17 cells and the expression of associated transcription factors and cytokines in the peripheral blood of patients with HBV-ACLF in early, middle, and late disease stages. The overall aim of the study was to investigate the regulatory role of the Treg/Th17 axis at different stages of viral- and endotoxin-mediated inflammation during HBV-ACLF, thereby providing new directions for the clinical treatment of liver failure.

## 2. Materials and Methods

### 2.1. Patients

A total of 60 patients with HBV-ACLF, admitted to the First Hospital of Hunan University of Chinese Medicine, China, between February 2018 and December 2018, were enrolled, including early-stage (*n* = 20), middle-stage (*n* = 20), and advanced-stage patients (*n* = 20). Twenty patients with chronic hepatitis B (CHB) on the immunoactive phase (including HBeAg-negative CHB or HBeAg-positive CHB) and 20 healthy individuals were recruited as controls. All patients were aged between 18 and 65 years and were negative for hepatitis A, C, D, E, or HIV infections. Enrolled patients did not have hepatocellular carcinoma or any extrahepatic malignancy. None of the patients had history of alcohol abuse, hepatotoxic drug use, chronic autoimmune, metabolic, renal, cardiac, or pulmonary diseases, nor had received antiviral treatment or immunosuppressive therapy during the previous 3 months. The study protocol was approved by the Ethics Committee of the First Hospital of Hunan University of Chinese Medicine (No. PJ20170720), and written informed consent was obtained from all participants.

### 2.2. Diagnostic Criteria of ACLF Staging

Early-stage ACLF was described as follows: serum bilirubin ≥ 171 *μ*mol/L, prothrombin activity (PTA) < 40% or international normalized ratio (INR) ranging from 1.5 to 1.9, no hepatic encephalopathy (HE), or other complications. Middle-stage ACLF was described as follows: serum bilirubin ≥ 171 *μ*mol/L, PTA within 20–30% or international normalized ratio (INR) ranging from 1.9 to 2.6, I-II HE and/or ascites, and infection. Late-stage ACLF was described as follows: serum bilirubin ≥ 171 *μ*mol/L, PTA < 20% or international INR > 2.6, and III–IV HE (or hepatorenal syndrome, gastrointestinal bleeding, and severe infection).

### 2.3. Prognostic Criteria for HBV-ACLF Patients

Patients with HBV-ACLF (*n* = 60) were divided into improvement (*n* = 43) and deterioration (*n* = 17) groups based on their condition at discharge or occurrence of death at the hospital. In the improvement group, symptoms and signs of liver failure disappeared or improved significantly, with a considerable improvement in liver function indexes (serum bilirubin and alanine aminotransferase decreased by over 50%). In the deterioration group, symptoms and signs did not improve, and deterioration was observed, along with organ failure, worsening of other vital signs, or death after ineffective treatment.

### 2.4. Clinical Data Collection

Demographic data, laboratory measurements (e.g., WBC count, lymphocyte (LYM) count, serum albumin (ALB), total bilirubin (TBil), alanine aminotransferase (ALT), aspartate aminotransferase (AST), PTA, prothrombin time (PT), INR, and creatinine levels), and information on HBV infection biomarker levels, virus infection time, and HBV DNA levels were collected. Laboratory data, HBV infection biomarkers, and HBV DNA levels were obtained at week 0.

### 2.5. Flow Cytometry

Peripheral blood mononuclear cells (PBMCs) were isolated from 2 mL of fresh heparinized blood via Ficoll-Hypaque (4A Biotech, Beijing, China) density gradient centrifugation. RPMI1640 (150 *μ*L) and cell stimulation cocktail (0.5 *μ*L) were added to the isolated PBMCs, evenly mixed, and incubated in a 37°C water bath for 6 h. The activated PBMCs were then incubated with anti-CD4, anti-CD8, anti-CD3, and anti-CD25 antibodies (Becton, Dickinson and Company, San Jose, CA, USA) at room temperature for 20 min. Anti-IL-17A (5 *μ*L) and anti-FoxP3 (5 *μ*L) antibodies were then added, followed by incubation at room temperature for 30 min. After staining, the cells were washed twice with PBS and fixed in 1% paraformaldehyde. The stained cells were subsequently processed on an Accuri C6 FCM (Becton, Dickinson and Company) for analysis. All data were analyzed using the Accuri C6 FCM software.

### 2.6. Cytometric Bead Array (CBA)

Blood samples for the measurement of cytokines were collected early in the morning. The CBA assay (CBA immunoassay kit; Becton, Dickinson and Company) was used for quantitative determination of serum cytokines (Human Inflammatory Kit), according to the manufacturer's instructions. The Inflammatory CBA Kit comprises microbeads coupled to mAbs against IL-6, IL-10, TGF-*β*, IL-17A, and IL-23. A secondary phycoerythrin-labeled anti-cytokine antibody was used, and the concentration of individual cytokines was determined based on fluorescence intensity. Data were acquired on a FACSVerse flow cytometer (Becton, Dickinson and Company). Sample analysis was performed using BD FCAP Array 3.0 software (Becton, Dickinson and Company).

### 2.7. Quantitative Real-Time PCR (qRT-PCR)

Total RNA was extracted from PBMCs using the Redzol reagent (Invitrogen, Waltham, MA, USA) according to the manufacturer's protocol. The extracted RNA was reverse-transcribed to first-strand cDNA using the Transcriptor First Strand cDNA Synthesis Kit (Promega, Madison, WI, USA). Gene expression was quantified on an ABI 7500 thermal cycler (Applied Biosystems, Waltham, MA, USA) using the SYBR Green PCR Master Mix Kit (Promega). The amplification program was as follows: initial denaturation at 94°C for 5 min, followed by 40 cycles at 94°C for 1 min, 58°C for 1 min, 72°C for 1 min, and 83°C for 1 min. *β*-Actin was used as internal control. The primer sequences used are shown in Table 1. For each sample, PCR was performed twice with triplicates for each sample, and the data were analyzed using the thermal cycler software to calculate the *Δ*Ct value.

### 2.8. Model for End-Stage Liver Disease Score (MELD Score)

The MELD score was determined as per the following equation:

MELD score = 3.78 × ln [TBIL (mg · dL^−1^)] + 11.2 × ln INR + 9.57 × ln [creatinine (mg · dL^−1^)] + 6.43 × (etiology : 0 if cholestatic or alcoholic, 1 otherwise).

### 2.9. Statistical Analysis

Statistical analyses were performed using the SPSS software (ver. 25.0; IBM Corp., Armonk, NY, USA), and the figures were produced using Prism 8.0 (GraphPad Software, San Diego, CA, USA). All data are expressed as the mean ± standard deviation or median (minimum and maximum) for continuous variables and as a number (percentage) for categorical variables. Differences between three or more groups were analyzed using one-way ANOVA, and LSD multiple comparison was used for comparison between groups. The correlations of CRP, PCT, and LPS with Th17 and Treg frequency were determined via Pearson's or Spearman's correlation analyses. A two-sided *P* value < 0.05 was considered to indicate statistical significance.

## 3. Results

### 3.1. Participant Characteristics

In total, 60 HBV-ACLF patients, 20 CHB patients, and 20 healthy controls (NC) were included in the study as per the inclusion and exclusion criteria. The number of male participants in each group was greater. There were no significant differences in the percentage of hepatitis B, antigen (HBeAg) positivity, and HBV DNA quantity among the early-stage ACLF (ACLF-E), mid-stage ACLF (ACLF-M), late-stage ACLF (ACLF-L), and CHB patients. There were significant differences in WBC and LYM counts, and serum ALB levels between all groups. WBC count in the ACLF-L group was significantly higher than in the NC and CHB groups (*P* < 0.05). LYM count and ALB levels in HBV-ACLF patients were significantly lower than those in the NC and CHB groups (*P* < 0.05). Furthermore, LYM count and ALB levels in HBV-ACLF patients decreased with disease progression. There was no significant difference in the levels of ALT, AST, and TBil between the ACLF-E, ACLF-M, and ACLF-L groups. In contrast, significant differences in PT, INR, and MELD scores were observed between these groups. The demographic and clinical characteristics of the participants are shown in Table 2.

### 3.2. Treg and Th17 Frequencies Increase with the Progression of ACLF

**Figure 1:**
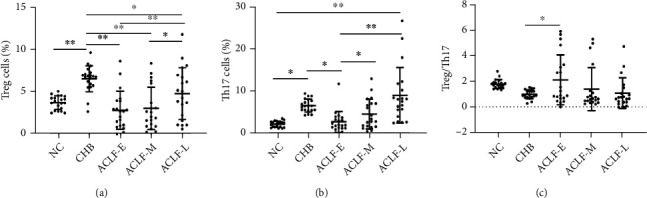
Frequency of T-regulatory (Treg) and T-helper (Th17) cells, and analysis of the Treg/Th17 ratio in peripheral blood mononuclear cells. (a) Frequency of Treg cells in early-stage (ACLF-E), mid-stage (ACLF-M), and late-stage (ACLF-L) patients with acute-on-chronic liver failure (ACLF), as well as in chronic hepatitis B (CHB) and healthy (NC) control groups. (b) Frequency of Th17 cells in ACLF-E, ACLF-M, ACLF-L, CHB, and NC groups. (c) Changes in Treg/Th17 ratio. Significant *P* values are indicated (^∗^*P* < 0.05; ^∗∗^*P* < 0.01).

**Figure 2:**
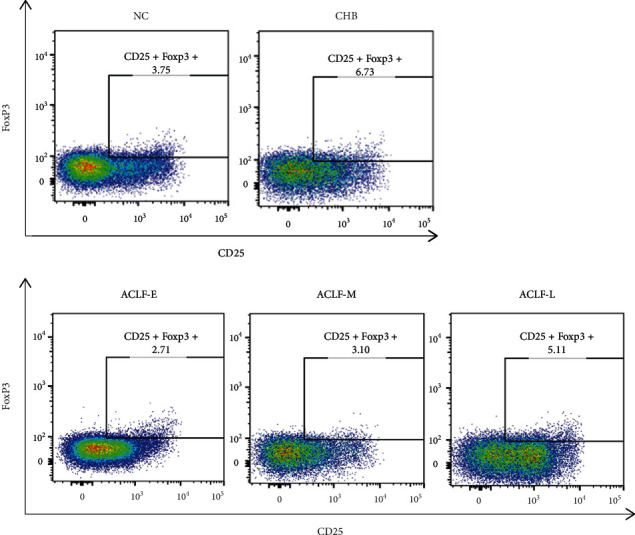
Flow cytometry analysis of the frequency of peripheral blood T-regulatory cells (CD4^+^CD25^+^FoxP3^+^) in each group. The plots show representative data of three independent analyses. NC: normal control; CHB: chronic hepatitis B; ACLF-E: early-stage ACLF; ACLF-M: mid-stage ACLF; ACLF-L: late-stage ACLF; ACLF: acute-on-chronic liver failure.

**Figure 3:**
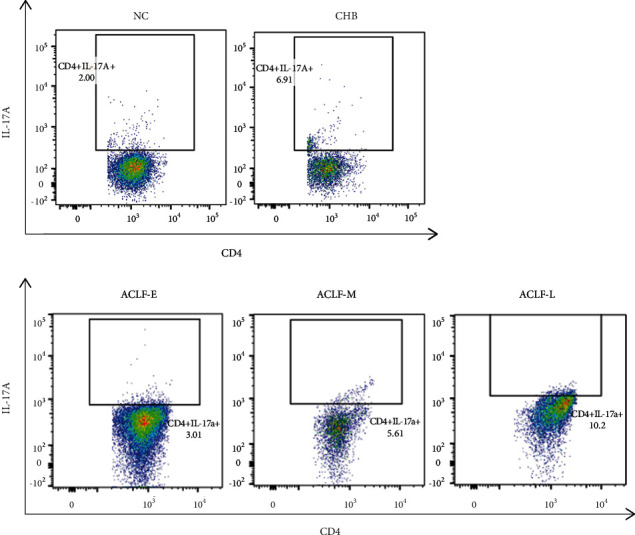
Flow cytometry analysis of the frequency of peripheral blood T-helper 17 cells (CD4^+^IL-17A^+^) in each group. The plots show representative data of three independent analyses. NC: normal control; CHB: chronic hepatitis B; ACLF-E: early-stage ACLF; ACLF-M: mid-stage ACLF; ACLF-L: late-stage ACLF; ACLF: acute-on-chronic liver failure.

We assessed the frequency of Treg and Th17 cells by flow cytometry and calculated the Treg/Th17 ratio. The frequency of Th17 cells in the ACLF-E, ACLF-M, and ACLF-L groups was significantly higher than in patients with CHB or normal controls. The frequencies of both cell populations gradually increased with the progression of ACLF, from early through late-stage disease. A significant difference between the late- and early-stage groups (*P* < 0.05, *P* < 0.01) was observed. Moreover, a significant difference was also observed between the late- and middle-stage groups (*P* < 0.05). Frequency was positively correlated with the severity of ACLF. Overall, Th17 cells were more abundant than Treg cells, resulting in a decrease of the Treg/Th17 ratio with disease progression (Figures 1–3).

### 3.3. Serum IL-10, TGF-*β*, TNF-*α*, IL-17A, and IL-23 Levels Increase with the Progression of ACLF

**Figure 4:**
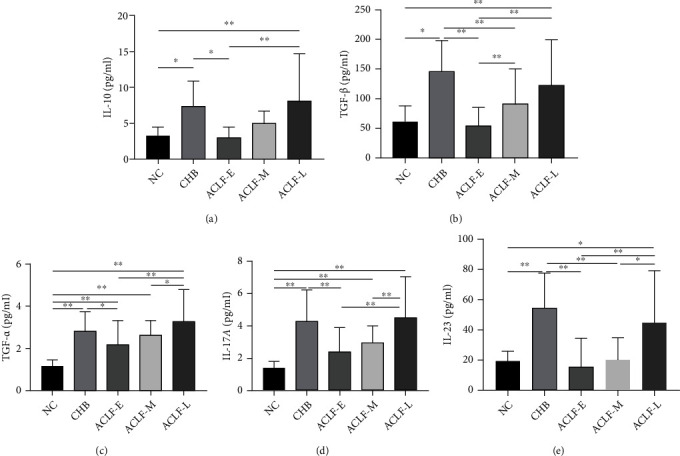
Serum levels of (a) IL-10, (b) TGF-*β*, (c) TNF-*α*, (d) IL-17A, and (e) IL-23 in all study participants. Significant *P* values are indicated (^∗^*P* < 0.05; ^∗∗^*P* < 0.01). IL-10: interleukin-10; TGF-*β*: transforming growth factor-*β*; IL-17A: interleukin-17A; TNF-*α*: tumor necrosis factor-*α*; IL-23: interleukin-23; NC: normal control; CHB: chronic hepatitis B; ACLF-E: early-stage ACLF; ACLF-M: mid-stage ACLF; ACLF-L: late-stage ACLF; ACLF: acute-on-chronic liver failure.

CBA was performed to quantify the serum levels of cytokines IL-10, TGF-*β*, TNF-*α*, IL-17A, and IL-23 in each participant. Compared with the NC group, the levels of IL-10 and TGF-*β* were lower in the ACLF-E group, whereas TNF-*α* and IL-17A were higher. The serum levels of all cytokines were significantly higher in the ACLF-M and ACLF-L groups when compared to those in the NC group. Moreover, the levels of IL-10, TGF-*β*, TNF-*α*, IL-17A, and IL-23 were significantly lower in the ACLF-E and ACLF-M groups than those in the CHB group (*P* < 0.05, *P* < 0.01). The serum levels of Treg-secreted cytokines IL-10 and TGF-*β* gradually increased with ACLF progression from early- to mid- and late-stage disease (*P* < 0.05, *P* < 0.01). Furthermore, the levels of TNF-*α*, IL-17A, and IL-23 secreted by Th17 cells gradually increased, with a significant difference between the ACLF-L and ACLF-E groups (*P* < 0.05, *P* < 0.01) (Figure 4).

### 3.4. FoxP3 Expression Decreases with the Progression of ACLF, Whereas That of ROR-*γ*t Increases

**Figure 5:**
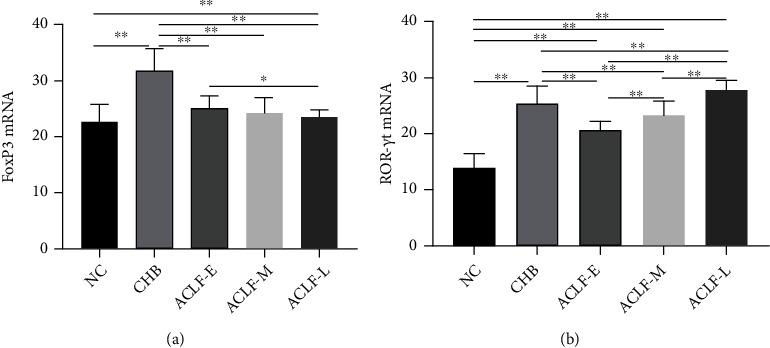
Peripheral blood (a) *FoxP3* and (b) *ROR-γt* expression in all study participants. Significant *P* values are indicated (^∗^*P* < 0.05; ^∗∗^*P* < 0.01). FoxP3: transcription factor forkhead box P3; *ROR-γt*: retinoid-related orphan nuclear receptor *γ*t; NC: normal control; CHB: chronic hepatitis B; ACLF-E: early-stage ACLF; ACLF-M: mid-stage ACLF; ACLF-L: late-stage ACLF; ACLF: acute-on-chronic liver failure.

qRT-PCR was performed to determine the expression Treg-specific transcription factor *FoxP3* and Th17-specific transcription factor *ROR-γt* in peripheral blood. Compared with the NC group, *FoxP3* and *ROR-γt* were significantly upregulated in the CHB, ACLF-E, ACLF-M, and ACLF-L groups. *FoxP3* expression was highest in the CHB group, whereas that of *ROR-γt* was highest in the ACLF-L group. The expression of *FoxP3* gradually decreased with ACLF progression, with the difference between the ACLF-L and ACLF-E groups being statistically significant (*P* < 0.05). In contrast, the levels of *ROR-γt* gradually increased with disease progression, with significant differences among the three groups (*P* < 0.01) (Figure 5).

### 3.5. CRP, PCT, and LPS Levels Increase with ACLF Progression

**Figure 6:**
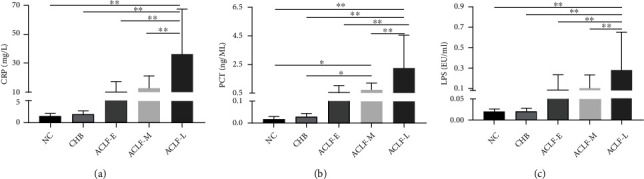
Serum levels of (a) CRP, (b) PCT, and (c) LPS in all study participants. Significant *P* values are indicated (^∗^*P* < 0.05; ^∗∗^*P* < 0.01). CRP: c-reactive protein; PCT: procalcitonin; LPS: lipopolysaccharide; NC: normal control; CHB: chronic hepatitis B; ACLF-E: early-stage ACLF; ACLF-M: mid-stage ACLF; ACLF-L: late-stage ACLF; ACLF: acute-on-chronic liver failure.

The dysregulated inflammatory response is a major pathological mechanism in ACLF. Inflammatory mediators are strongly associated with the severity of ACLF [16]. The serum levels of CRP, PCT, and LPS were determined in all subject groups, revealing that all increased with the progression of ACLF. Significant differences were observed between the ACLF-E and ACLF-L groups as well as between the ACLF-M and ACLF-L groups (*P* < 0.01) (Figure 6).

### 3.6. CRP, PCT, and LPS Serum Levels Are Positively Correlated with Th17, but Not with Treg Frequency

**Figure 7:**
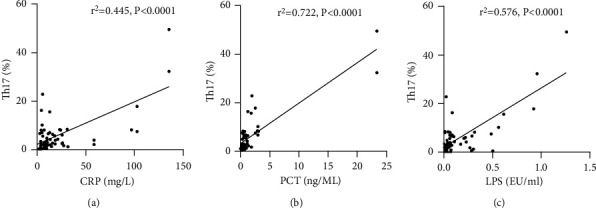
Correlation of (a) CRP, (b) PCT, and (c) LPS serum levels with Th17 frequency in patients with HBV-ACLF. CRP: c-reactive protein; PCT: procalcitonin; LPS: lipopolysaccharide; Th17: T-helper 17 cells; HBV-ACLF: hepatitis B virus-related acute-on-chronic liver failure.

**Figure 8:**
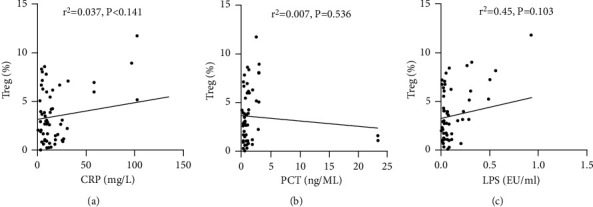
Correlation of CRP, PCT, and LPS levels with Treg frequency in patients with HBV-ACLF. CRP: c-reactive protein; PCT: procalcitonin; LPS: lipopolysaccharide; Th17: T-helper 17 cells; HBV-ACLF: hepatitis B virus-related acute-on-chronic liver failure.

We analyzed the correlation of CRP, PCT, and LPS levels with Th17 and Treg frequency. The serum levels of all three inflammatory biomarkers were positively correlated with Th17 frequency in all HBV-ACLF patients (Figure 7). In contrast, serum CRP, PCT, and LPS exhibited no correlation with Treg levels (Figure 8).

### 3.7. Binary Logistic Regression Analysis of ACLF Risk Factors

**Figure 9:**
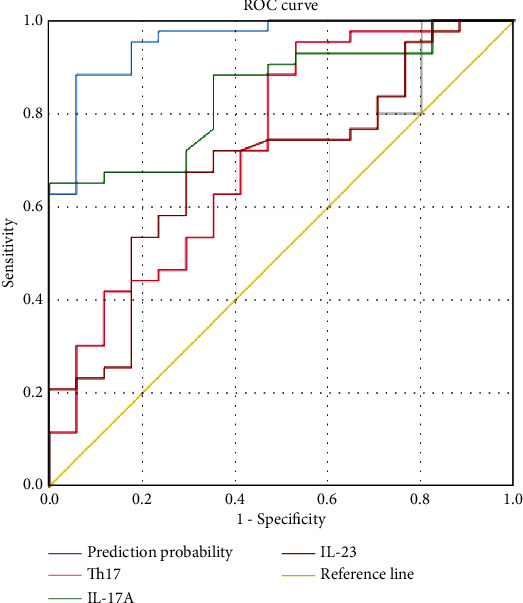
Receiver operating characteristic (ROC) curve of the predictive model and independent risk factors.

Among the 60 patients with HBV-ACLF included in the study, 43 showed symptom improvement and 17 experienced disease aggravation. The correlation between the frequency of Treg and Th17 cells, as well as IL-10, TGF-*β*, TNF-*α*, IL-17A, IL-23, FoxP3, and ROR-*γ*t levels with ACLF prognosis, was analyzed using binary logistic regression (Table 3). The results indicated that Th17 (*P* < 0.05, odd ratio (OR) = 1.221, 95% confidence interval (CI) = 1.034–1.441), IL-17A (*P* < 0.01, OR = 3.429, 95%CI = 1.394–8.435), and IL-23 (*P* < 0.05, OR = 1.066, 95%CI = 1.003–1.134) levels were independent risk factors for ACLF. The logistic regression model was established as follows: logistic (*P*) = −6.553 + 0.199 × Th17 + 1.232 × IL‐17A + 0.064 × IL‐23. The Hosmer-Lemeshow test was used to assess goodness of the fit in the logistic regression model (*x*2 = 4.637, *P* = 0.796). A good fit was observed, and the logistic regression model was statistically significant. Receiver operating characteristic curve analysis revealed an area under the curve of 0.956, which was higher than that of any independent risk factor. The maximum Youden index value was 0.825, with a corresponding sensitivity and specificity of 88.4% and 94.1%, respectively (Figure 9). The 60 patients with HBV-ACLF were selected as a validation cohort. With a prediction probability of 0.5 as threshold, the total correct predication rate was 90.00% (54/60), the positive predictive value was 93.00% (43/40), and the negative predictive value was 82.4% (14/17).

## 4. Discussion

Recent studies have shown that immune activation plays a major role in the occurrence and development of liver failure. Persistent inflammatory response and immune dysregulation are considered core mechanisms in HBV-ACLF pathophysiology [4, 5] that determine the severity of tissue damage and patient prognosis. Therefore, the identification of novel targets for immunosuppression and to restore the immune balance based on the immune profile of patients at different disease stages is a major goal within HBV-ACLF research. In the current study, we confirmed that the frequencies of Treg and Th17 cells in peripheral blood increase with HBV-ACLF progression. These cell populations were previously described as closely associated with the occurrence and development of HBV-ACLF [17–19]. When compared with Tregs, Th17 cells increased to a greater extent in ACLF patients, and the two CD4^+^ T cell subsets were positively correlated with each other.

The close relationship between Th17 and Treg cells has been acknowledged in recent years as they share a common developmental pathway, with TGF-*β* being necessary for the differentiation of both. Th17 cells are activated by TGF-*β*, whereas Treg cells are activated by TGF-*β* as well as IL-10 [20–22]. Th17 cells are potent proinflammatory effectors, participating in liver injury and viral clearance after HBV infection. Studies have reported that IL-17A levels and Th17 cell frequency in the peripheral blood of patients with CHB are positively correlated with liver injury. Circulating Th17 cells mainly accumulate within the liver of CHB patients, and their frequency gradually increases during progression from CHB to ACLF [23]. Treg cells play an important role in inducing and maintaining immune tolerance and terminating the immune response. Herein, a Treg/Th17 imbalance was observed in patients with CHB and HBV-ACLF. Consistent with previous studies, we confirmed that Th17 and Tregs in the peripheral blood of CHB and HBV-ACLF patients were significantly increased compared with healthy controls. Furthermore, the highest Treg frequency was observed in patients with active CHB, whereas Th17 cells were most abundant in HBV-ACLF patients. Previous studies have shown that Treg cell-mediated immunosuppression mainly occurs during the CHB stage, whereas Th17-mediated inflammatory damage is sustained throughout the HBV-ACLF stage [23]. Although Th17 and Treg cells are known to be significantly increased in ACLF patients, our understanding of their frequency and interaction throughout the different disease stages is limited.

In the current study, we observed that the increase of Th17 cell frequency in CHB and HBV-ACLF patients occurred concurrently to an increase in Treg cell frequency. Noteworthy, Th17 frequency in ACLF patients was higher than that of Tregs. More importantly, the Treg/Th17 ratio gradually decreased with the progression of disease. These data highlight the involvement of both immune cell populations in HBV-ALCF. Furthermore, dynamic shifts in both are important for the maintenance of immune balance and for avoiding tissue damage. During HBV-ACLF pathogenesis, inflammation gradually transitions from an antiviral response during the early and middle stages to an endotoxin-mediated inflammatory response in the late disease stages. During HBV-mediated immune injury, immune hyperactivation is observed in ACLF patients. Moreover, the Treg/Th17 balance is dysregulated [24], and aberrant release of Th17 cytokines (IL-17, IL-23, and TNF-*α*) is observed, resulting in a proinflammatory state. In addition, Treg cells and their effector cytokines TGF-*β* and IL-10 are also upregulated via compensatory induction. However, their anti-inflammatory counterbalance effect is insufficient to effectively inhibit the aberrant inflammatory response. LPS-driven inflammation plays a major role during the late stage of ACLF. Intestinal mucosal permeability is increased, allowing the entry of gut microbes into circulation, which in turn leads to LPS-induced intestinal endotoxemia [25, 26]. Furthermore, the Treg/Th17 imbalance worsens, with a significant increase in Th17 frequency, and consequent release of effector cytokines IL-17, IL-23, and TNF-*α* will in turn exacerbate the immune response. Herein, CRP, PCT, and LPS levels were also found to be increased and positively correlated with Th17 frequency. The risk of infection is also considerably greater in these patients; thus, infection may be a major factor aggravating the course of ACLF [27]. Taken together, the observed increase in Treg levels during ACLF may be a negative feedback effect for counterbalancing the Th17 response. However, the greater increase in Th17 cells overwhelms this homeostatic response, driving uncontrolled inflammation and leading to end-stage liver disease.

FoxP3 and ROR-*γ*t are transcription factors specific to Treg and Th17 cells, respectively, and drive their differentiation. Moreover, high concentrations of TGF-*β* induce FoxP3 expression in immature CD4^+^ T cells and promote their differentiation into Treg cells [28]. In contrast, TGF-*β* along with IL-6 or IL-21 can induce ROR-*γ*t expression and promote subsequent differentiation of Th17 cells [29]. In turn, IL-21 inhibits FoxP3 expression and promotes Th17 cell differentiation by modulating TGF-*β* signal transduction [30]. Some studies have shown that increased FOXP3 expression is involved in the pathogenesis of CHB [31]. In addition, FoxP3 and ROR-*γ*t levels were herein found to be significantly higher in HBV-ACLF patients compared with healthy and CHB subjects, suggesting that the expression of both transcription factors is related to the severity of disease [14, 17]. Hence, Treg and Th17 cells in patients with HBV-ACLF increase with the progression of the disease through the early, middle, and late disease stages. Furthermore, the imbalanced Treg/Th17 was found to shift toward Th17, which agrees with previous findings. In the peripheral blood of patients with HBV-ACLF, ROR-*γ*t expression changed concurrently with the Th17 cell frequency, whereas FoxP3 expression did not change in concert with the Treg levels. Interestingly, FoxP3 expression began to gradually decrease with an increase in Tregs, and no definite correlation was observed between the two. Recent studies have shown that Treg cells can downregulate FoxP3 expression under certain conditions, thus losing their immunosuppressive function [32]. The key factor leading to FoxP3 downregulation is the high level of inflammatory cytokines within the surrounding environment, significantly suppressing Treg function and preventing the Treg-mediated inhibition of inflammation. In contrast, here, we found that ROR-*γ*t expression was positively associated with Th17 dominance. Thus, it is possible that FoxP3 expression is influenced by other genes, as well as differences between the liver and the blood microenvironment.

Finally, we designed a prognostic prediction model for HBV-ACLF based on the herein collected Treg- and Th17-related data. Currently, only few studies have provided HBV-ACLF prognostic models. Binary logistic regression analysis indicated that Th17, IL-17A, and IL-23 are independent risk factors for ACLF. However, Treg cells and related cytokines were not significantly correlated to disease progression, and the relationship between Treg cells and prognosis of ACLF remains unclear. To the best of our knowledge, there is no consensus on whether Treg cells can effectively control the Th17 response in human disease. Importantly, the Treg/Th17 ratio was associated with ACLF severity. The prognostic model established herein furthers our understanding on the relationship between Th17 and Tregs in HBV-ACLF, as well as their association with disease prognosis. These results suggest that Th17 cells and the Treg/Th17 axis may represent candidates for further study in ACLF, especially in orthotopic liver transplantation.

This study is aimed at investigating shifts in Treg/Th17 balance and both pro/anti-inflammatory cytokine level expression in the peripheral blood of HBV-ACLF patients at different disease stages. However, there are some limitations that compromise this study. First, this study only detected the peripheral blood lymphocyte frequency of the subjects, excluding the same lymphocyte population frequency in concomitant biopsy samples. This may not be sufficient to reflect what is seen in the liver. In addition, this is a single-center study. Due to the small sample size, the conclusion may have limited generalizability. Therefore, these conclusions need more cases to be verified.

## 5. Conclusion

In conclusion, the Treg/Th17 cell imbalance occurs throughout all stages of HBV-ACLF pathogenesis. The frequency of Th17 cells and the levels of their effectors (IL-17A, IL-23, and TNF-*α*) were upregulated with disease progression, resulting in a proinflammatory reaction. In parallel, a compensatory induction of Treg cells and their effector cytokines (TGF-*β* and IL-10) was also observed, yet an imbalance toward Th17 signals remained. During late-stage endotoxin-driven inflammation, the Treg/Th17 imbalance was further aggravated. Th17 cells, IL-17A, and IL-23 may represent valuable independent risk factors for the prognosis of HBV-ACLF. The current findings provide new insight into the pathogenesis of HBV-ACLF and its clinical prognosis.

## Figures and Tables

**Table 1:** Primer sequences used in the study.

Gene	Sequence	Size (bp)
*FoxP3*	5′-AAGAACGCCATCCGCCACAAC-3′	92
	5′-TCCAGCTCATCCACGGTCCAC-3′	92

*ROR-γt*	5′-AGCGGCAACAGCAGCAACAG-3′	132
	5′-CAGGCAGGTCAGGCGAGGAG-3′	132

*ACTB*	5′-GCACTCTTCCAGCCTTCCTTCC-3′	93
	5′-GCGGATGTCCACGTCACACTTC-3′	93

**Table 2:** Baseline demographic and clinical characteristics of all participants.

	NC (*n* = 20)	CHB (*n* = 20)	ACLF-E (*n* = 20)	ACLF-M (*n* = 20)	ACLF-L (*n* = 20)
Age (mean (SD), years)	31.30 ± 5.09	44.75 ± 13.02	44.75 ± 11.92	45.05 ± 8.86	45.1 ± 12.22
Sex (male/female)	16/4	17/3	18/2	17/3	16/4
Virus infection time					
<10 years	N.D	4	0	3	1
≥10 years	N.D	16	20	17	19
HBeAg (+/−)	N.D	9/11	6/14	7/13	4/16
HBV-DNA (×10^7^IU/mL)	N.D	2.2 ± 7.41	1.2 ± 3.16	8.3 ± 34.80	7.9 ± 20.81
WBC (×10^9^/L)	5.84 ± 0.69	5.12 ± 1.32	6.64 ± 3.53	6.43 ± 4.63	8.18 ± 3.67
LYM (%)	35.59 ± 2.18	29.15 ± 4.13	21.46 ± 7.51	20.36 ± 6.83	14.81 ± 7.80
ALB (g/L)	47.24 ± 2.79	41.69 ± 3.78	35 ± 3.21	33.96 ± 8.82	33.07 ± 6.08
ALT (IU/L)	22.14 ± 5.64	134.70 ± 154.87	370.86 ± 425.01	287.31 ± 421.69	356.85 ± 606.74
AST (IU/L)	19.95 ± 7.24	186.81 ± 229.78	370.82 ± 767.01	197.92 ± 250.69	304.84 ± 569.32
TBil (*μ*mol/L)	13.61 ± 3.18	11.25 ± 6.24	304.43 ± 142.01	298.35 ± 178.82	360.59 ± 201.53
PT (S)	N.D	12.53 ± 1.17	19.15 ± 1.90	25.67 ± 2.87	34.60 ± 11.10
INR	N.D	1.02 ± 0.10	1.58 ± 0.17	2.12 ± 0.25	2.92 ± 1.01
MELD score	N.D	N.D	21.27 ± 3.29	24.45 ± 2.36	28.47 ± 3.11

Data are presented as numbers (%) or mean ± SD, unless otherwise indicated. NC: normal control; CHB: chronic hepatitis B; ACLF-E: early-stage ACLF; ACLF-M: mid-stage ACLF; ACLF-L: late-stage ACLF; HBeAg: hepatitis B e antigen; HBV: hepatitis B virus; WBC: white blood cell; LYM: lymphocyte; ALB: albumin; ALT: alanine aminotransferase; AST: aspartate minotransferase; TBil: total bilirubin; PT: prothrombin time; INR: international normalized ratio; MELD score: Model for End-Stage Liver Disease score; ACLF: acute-on-chronic liver failure; N.D.: not determined.

**Table 3:** Binary logistic regression analysis of risk factors for acute-on-chronic liver failure.

Factor	*B*	SE	Wald	Sig	OR	95% CI for OR	
						Lower	Upper
Treg	0.238	0.199	1.430	0.232	1.269	0.859	1.875
Th17	0.199	0.085	5.562	0.018	1.221	1.034	1.441
IL-10	-0.297	0.316	0.878	0.349	0.743	0.400	1.382
TGF-*β*	0.007	0.013	0.312	0.576	1.007	0.982	1.033
TNF-*α*	0.447	0.387	1.335	0.248	1.563	0.733	3.334
IL-17A	1.232	0.459	7.201	0.007	3.429	1.394	8.435
IL-23	0.064	0.031	4.204	0.040	1.066	1.003	1.134
FoxP3	-0.214	0.296	0.521	0.470	0.807	0.452	1.443
ROR-*γ*t	0.064	0.195	0.108	0.743	1.066	0.728	1.561
Constant	-6.553	10.235	0.410	0.522	0.001		

*B*: partial regression coefficient; SE: standard error; Sig: significance; OR: odds ratio; CI: confidence interval.

## Data Availability

The data used to support the findings of this study are available from the first author or corresponding author upon request.
